# Infant mortality and morbidity associated with preterm and small-for-gestational-age births in Southern Mozambique: A retrospective cohort study

**DOI:** 10.1371/journal.pone.0172533

**Published:** 2017-02-17

**Authors:** Alberto L. García-Basteiro, Llorenç Quintó, Eusebio Macete, Azucena Bardají, Raquel González, Arsenio Nhacolo, Betuel Sigauque, Charfudin Sacoor, María Rupérez, Elisa Sicuri, Quique Bassat, Esperança Sevene, Clara Menéndez

**Affiliations:** 1 Centro de Investigação em Saude de Manhiça, Manhiça, Maputo Province, Mozambique; 2 ISGlobal, Barcelona Ctr. Int. Health Res. Hospital Clínic—Universitat de Barcelona, Barcelona, Spain; 3 Amsterdam Institute for Global Health and Development, Academic Medical Centre, Amsterdam, The Netherlands; 4 Consorcio de Investigación Biomédica en Red de Epidemiología y Salud Pública, Barcelona, Spain; 5 School of Public Health, Imperial College London, London, United Kingdom; Centre Hospitalier Universitaire Vaudois, FRANCE

## Abstract

**Background:**

Preterm and small for gestational age (SGA) births have been associated with adverse outcomes during the first stages of life. We evaluated the morbidity and mortality associated with preterm and SGA births during the first year of life in a rural area of Southern Mozambique.

**Methods:**

This is a retrospective cohort study using previously collected data from children born at the Manhiça District Hospital in two different periods (2003–2005 and 2010–2012). Newborns were classified as being preterm and/or SGA or as babies not fulfilling any of the previous conditions (term non-SGA). All children were followed up for a year for morbidity and mortality outcomes.

**Results:**

A total of 5574 live babies were included in the analysis. The prevalence of preterm delivery was 6.2% (345/5574); the prevalence of SGA was 14.0% (776/5542) and 2.2% (114/5542) of the children presented both conditions. During the neonatal period, preterm delivery and SGA were associated with 13 (HR: 13.0, 95% CI 4.0–42.2) and 5 times (HR: 4.5, 95% CI: 1.6–12.6) higher mortality compared to term non SGA babies. Risk of hospitalization was only increased when both conditions were present (IRR: 3.5, 95%CI: 1.5–8.1). Mortality is also increased during the entire first year, although at a lower rate.

**Conclusions:**

Neonatal and infant mortality rates are remarkably high among preterm and SGA babies in southern Mozambique. These increased rates are concentrated within the neonatal period. Prompt identification of these conditions is needed to implement interventions aimed at increasing survival of these high-risk newborns.

## Introduction

Preterm birth is the world’s leading cause of death in children under five years[1]. It has been estimated that each year, 11% of all deliveries in the world are premature, and one million out of six million child deaths are due to complications of prematurity[2,3]. Small for gestational age (SGA) births, are also a prevalent condition among newborns from low and middle income countries (up to 27% of all deliveries are SGA), with higher prevalence in South East Asia and Sahelian countries[4]. Preterm and SGA births are associated with adverse health consequences, including increased neonatal and infant mortality, childhood malnutrition, visual and hearing problems, and adulthood metabolic disease[5,6].

Both preterm birth and SGA are intrinsically associated with low birth weight and are not mutually exclusive. On the one hand, preterm birth is associated with multiple maternal and/or foetal conditions, including maternal and neonatal infections, vascular disease, uterine overdistension, pre-eclampsia/eclampsia or intrauterine growth restriction (IUGR)[7]. On the other hand, SGA is frequently associated with disorders such as foetal genetic/chromosomal defects or also to IUGR[8]. The latter is associated with factors that prevent normal circulation across the placenta causing poor nutrient and oxygen supply to the foetus, including maternal undernutrition, anemia, malaria, HIV and other acute or chronic infections[9]. Alternatively, SGA can result from an incorrect assessment of gestational age or a constitutionally–albeit not necessarily pathological- small size. However, since preterm and SGA babies are at risk of presenting different health problems they are associated with different morbidity and mortality risks[10]. Compared to SGA, preterm babies have been associated with higher risk of death during infancy, but lower risk of morbidity and better growth patterns during the first two years of life[10].

Despite the relative high prevalence and adverse outcomes associated with preterm births and SGA in low-income settings, very few studies have assessed their impact on neonatal and infant mortality and morbidity in sub Saharan Africa. Only one longitudinal study conducted in Malawi showed that preterm birth was associated with a greater risk of death as well as growth and development disabilities[11]. Understanding the true impact of these two common conditions is essential to improve pregnancy management and prevent their consequences in low income settings. Importantly, those regions with high rates of preterm births and low birth weight, mainly South East Asia and Africa, are also those with most fragile and underfinanced health programs, increasing the difficulties to tackle this health problem[12]. The main objective of this study was to evaluate the morbidity and mortality associated with preterm and SGA births during infancy in a rural area of Southern Mozambique.

## Methods

### Study setting

The study was conducted at the Centro de Investigacão em Saúde da Manhiça (CISM) in the District of Manhiça, a malaria endemic semi-rural area in Southern Mozambique. The CISM is adjacent to the Manhiça District Hospital (MDH) and runs a demographic surveillance system (DSS) covering 90000 inhabitants in 2010 in what constitutes the study area. A passive case detection system is also running at the HDM that covers all paediatric outpatient visits and admissions. More than 80% of the deliveries in the district are institutional[13]. The prevalence of HIV infection detected through the antenatal clinic (ANC) has steadily increased in recent years, ranging from 23.6% in 2003–2004[13] to 29.4% in 2010[14]. Infant and neonatal mortality rates varied from 83.9 and 26 in 2004 to 63.0 and 24·0 per 1000 live births in 2010 (Nhacolo A., Charfudin et al personal communication)[15]. Other health and demographic characteristics of the population of the district have been described elsewhere[16].

### Study design

This is a retrospective cohort study of collected data from children born at the MDH in two different time periods; period 1: from August 2003 to April 2005 and period 2: form March 2010 to March 2012. During these periods, gestational age was routinely captured for all births taking place at the MDH, due to the coexistence of research studies which required the assessment of gestational age. Infants were classified as being preterm and/or SGA, or as babies not fulfilling any of the previous conditions (term, non-SGA). All babies were followed up for a year for morbidity and mortality outcomes using the hospital passive case detection system and the DSS. Inclusion criteria for this analysis included living in the study area, being a live birth, being institutionally delivered, and having the gestational age and weight assessed at birth.

Gestational age was evaluated using two different methods based on postnatal examination of the newborn, namely, the Dubowitz test[17] (period 1) and the Ballard score[18] (period 2). Both methods are based on clinical assessment that includes neurological criteria on the infant’s maturity and other external physical criteria. Both methods are widely used in low-income countries, where ultrasound examination is not readily available. Dubowitz and Ballard’s tests were used in Manhiça due to the requirements of two different clinical trials evaluating antimalarials for prevention of malaria in pregnancy, which took place in those previously mentioned time periods[13,19,20]. Relevant socio-economic and demographic characteristics of the households of children included in the study are also recorded through the DSS.

### Case definitions and statistical methods

Neonatal mortality was defined as the death of a live born baby within the first 28 complete days after birth, and infant mortality as deaths occurring during the first 12 months of life. The post-neonatal period was defined as that comprised after day 28 and the last day of the first year of life (included). Preterm birth was defined as that occurring before the completion of 37 weeks of pregnancy. Low birth weight was defined as less than 2500 grams at birth[21]. Small-for-gestational-age (SGA) was defined as birth weight below the 10^th^ percentile for babies of the same gestational age[5]. Since no reference birthweight charts per percentile are available for the Mozambican population, we used as reference birthweights from a recent large study in HIV negative babies from Botswana[22].

Only live born babies (single and multiple deliveries) were included in this analysis. Incidence of outpatient visits or hospitalizations and mortality rates were calculated using time at risk from date of birth until date at one year of age, death or withdrawal. Mortality rates are expressed per 1000 children years at risk (CYAR). Association between risk factors and occurrence of any of the conditions at delivery was evaluated using univariate and multivariable logistic regression models. Hazard Ratio (HR) of mortality among different cohorts was evaluated using Cox Regression Models adjusted for child sex, HIV status of the mother, number of previous pregnancies, maternal age, period and socio economic status (SES). Incidence rate ratio (IRR) of outpatient clinic visits or hospitalizations was assessed using negative binomial regression due to the possibility of several episodes during the study period. Variables for the multivariate analysis in the logistic, Cox, and negative binomial regression models were selected using the forward-stepwise approach with a p-value lower than 0.1 (obtained through likelihood ratio test). Multivariable models were estimated by a complete case analysis with missing values removed. P values lower than 0.05 were considered statistically significant.

SES was calculated using Principal Component Analysis (PCA) following the methodology described elsewhere[23]. The families of the children were grouped into quintiles based on the SES rank.

All data were captured in handwritten CRFs and then double entered by data clerks into the OpenClinica software. Data analysis was performed using Stata 13 (Stata Corporation, College Station, TX, USA). Microsoft Excel (Microsoft Office Package 13) was used for building graphs and tables.

### Ethical considerations

This study is a retrospective analysis of previously collected information. Many participants were part of two research studies, whose protocols and informed consents were reviewed and approved by the National Ethics Board in Mozambique and the Ethics Committee from the Hospital Clinic of Barcelona (Spain)[13,19,20]. This specific study was approved by the Ethics Committee Hospital Clinic of Barcelona (Spain). Mothers/caregivers of children participating in the research studies signed a written informed consent form prior to enrolment. The study was conducted following the principles of the Declaration of Helsinki. The funding sources had no role in any step of the study, including the decision to submit the paper for publication.

## Results

### Prevalence of low birth weight, preterm delivery and SGA

A total of 5574 live babies with available data on gestational age were included in the analysis (3189 in period 1 and 2385 in period 2). Around 51.4% (2853/5554) of the babies were male and 29.0% (656/2265) were born to HIV infected mothers. Among all children included 26.6% (1397/5256) were born to primigravidae, and 23.2% (1219/5256 to women with more than 4 previous pregnancies.

The prevalence of low birth weight (<2500 g) in our sample was 10.3% (572/5570), that of very low birth weight (<1500 g) was 0.5% (29/5570), and the proportion of preterm delivery was 6.2% (345/5574). The prevalence of SGA was 14.0% (776/5542); among the SGA, 11.9% (662/5542) were at term SGA, while 2.1% (114/5542) of them fulfilled both definitions of preterm and SGA simultaneously. Nearly 4% [3.7% (205/5542)] of the infants were preterm but not SGA. Baseline characteristics of the study participants are depicted in **Table 1.**

**Table 1:** Baseline characteristics of participants included in the analysis. Columns are not mutually exclusive.

	Total live births*	Preterm n(%)	Total live births*	SGA n(%)	Total live births	Non preterm–non SGA n(%)	Total live births	Preterm & SGA n(%)
**Total**	5574	345(6.2)	5542	776 (14.0)	5542	4561 (82.3)	5542	114 (2.2%)
**Previous pregnancies**								
Primigravidae	1397	107 (7.7)	1390	295 (21.2)	1390	1035 (74.5)	1390	41 (3.0)
1–4 previous pregnancies	2640	147 (5.6)	2603	309 (11.8)	2623	2221 (84.7)	2623	41 (1.6)
>4	1219	59 (4.9)	1213	132 (10.0)	1213	1051 (86.4)	1213	23 (1.9)
**Mother's age**								
<20	1385	116 (8.4)	1373	300 (21.8)	1373	1007 (73.3)	1026	40 (2.9)
20–34	3290	166 (5.1)	3277	369 (11.3)	3277	2804 (85.6)	2466	52 (1.6)
>35	587	28 (4.8)	582	68 (11.7)	582	503 (86.4)	483	12 (2.1)
**Gestational age**								
<37	345	NA	319	114 (35.8)	319	NA	319	114(35.8)
37–42	5222	NA	5216	661 (12.7)	5216	4555 (87.3)	5216	NA
> 42	7	NA	7	1 (14.3)	7	6 (82.3)	7	NA
**Birthweight (mean)**								
<1500	29	20 (69.9)	24	24 (100.0)	24	0 (0.0)	24	15 (62.5)
1500–2500	572	201 (35.2	565	461 (81.6)	565	9 (1.6)	565	99 (17.6)
>2500	4969	123 (2.48)	4951	291 (5.9)	4951	4550 (91.9)	4951	0 (0.0)
**Newborn Sex**								
Male	2853	158 (5.54)	2833	342 (12.1)	2833	2408 (85.0)	2833	58 (2.1)
Female	2701	184 (6.81)	2689	431 (16.0)	2689	2137 (79.5)	2689	54 (2.1)
**Period**								
> 2003–2006	3189	187 (5.9)	3167	497(15.7)	3167	2563(80.9)	3167	60 (1.9)
< 2009–2012	2385	158 (6.6)	2375	279(11.8)	2375	377(84.1)	2375	54 (2.3)
**Study participant**								
No	3156	205 (6.5)	3135	437 (13.9)	3135	2571 (82.0)	3135	60 (1.9)
Yes	2418	140 (5.8)	2407	339 (14.1)	2407	1990 (82.7)	2407	54 (2.2)
**Mother's HIV status**								
Uninfected	1609	97 (6.0)	1605	218 (13.6)	1605	1337 (83.3)	1605	43(2.7)
Infected	656	38 (5.8)	651	94 (14.4)	651	532 (81.7)	651	11 (1.7)
**SES**								
Poorest	508	26 (5.1)	508	67 (13.2)	508	426 (83.9)	508	11 (2.2)
2nd quintile	495	30 (6.1)	492	60 (12.2)	492	413 (83.9)	492	8 (1.6)
3rd quintile	514	31 (6.1)	511	72 (14.1)	511	419 (82.0)	511	9 (1.8)
4th quintile	496	30 (6.1)	494	63 (12.8)	494	418 (84.6)	494	15 (3.0)
Wealthiest	503	22 (4.4)	500	56 (11.2)	500	432 (86.4)	500	9 (1.8)

NA: Not applicable.

* There were missing values for several variables. Those participants were not included in the analysis. The variable “mother’s HIV status” was only available for 2265 of the 5574 children with available gestational age and for 2256 of the 5542 with known SGA status.

Being multigravidae and older age were each associated with lower likelihood of SGA. If the mother had more than four previous pregnancies the odds of being SGA was 52% lower compared to the odds of primigravidae women (OR: 0.48, 95% CI: 0.25–0.90). Female sex and maternal HIV infection were also associated with being SGA (OR: 1.42 95% CI: 1.05–1.91 and OR: 1.78, 95% CI: 1.27–2.50, respectively). Likewise, the same variables were associated with preterm delivery, although without statistical significance in the multivariable model. **Tables 2 and 3** show the results of the univariate and multivariable logistic regression analysis after adjusting for potential confounders.

**Table 2:** Univariate and multivariable analysis of factors associated with preterm birth.

			Univariate Analysis		Multivariate analysis	
	Total	Preterm	uOR (95% CI)	p value*	aOR (95% CI)	p value^
		n (%)				
**Newborn Sex**						
Male	2491	83 (3.3)	1.00	<0.001		0.055
Female	2258	121 (5.4)	1.64 (1.23–2.18)		1.70 (0.98–2.95)	
**Previous pregnancies**						
Primigravidae	1095	60 (5.5)	1.00	0.006	1.00	0.66
1–4 previous pregnancies	2314	93 (4.0)	0.72 (0.52–1.01)		0.76 (0.34–1.71)	
>4	1081	30 (2.8)	0.49 (0.32–0.77)		0.60 (0.19–1.88)	
**Mother's age**						
<20	1073	66 (6.2)	1.00	<0.001	1.00	0.154
20–34	2908	104 (3.6)	0.57 (0.41–0.78)		0.46 (0.21–1.04)	
>35	514	11 (2.2)	0.33 (0.17–0.64)		0.31 (0.05–1.77)	
**Period**						
> 2003–2006	2670	107 (4.0)	1.00	0.260	1.00	0.40
< 2009–2012	2096	98 (4.7)	1.17 (0.89–1.55)		1.29 (0.71–2.35)	
**Study participant**						
No	2698	127 (4.7)	1.00	0.112	*#*	
Yes	2068	78 (3.8)	0.79 (0.59–1.06)			
**Mother's HIV status**						
Uninfected	1387	50 (3.6)	1.00	0.367	1.00	0.073
Infected	557	25 (4.5)	1.25 (0.76–2.05)		1.75 (0.95–3.21)	
**SES**						
Poorest	394	13 (3.3)	1.00	0.282	1.00	0.20
2nd quintile	454	21 (4.6)	1.42 (0.70–2.88)		1.50 (0.64–3.49)	
3rd quintile	457	17 (3.7)	0.13 (0.54–2.36)		1.04 (0.42–2.58)	
4th quintile	454	19 (4.2)	1.28 (0.62–2.63)		1.60 (0.68–3.74)	
Wealthiest	428	9 (2.1)	0.62 (0.26–1.48)		0.55 (1.18–1.69)	

*p value calculated through Wald Tests.

^p value calculated through LR test.

# variable excluded in the model due to high collinearity with mother’s HIV status.

uOR = unadjusted odds ratio.

aOR = adjusted odds ratio.

**Table 3:** Univariate and multivariable analysis of factors associated with small for gestational age (SGA).

			Univariate Analysis		Multivariate analysis	
		SGA	uOR (95% CI)	p value*	aOR (95% CI)	p value^
	Total	n (%)				
**Newborn Sex**						
Male	2692	284 (10.6)	1	<0.001	1	0.021
Female	2137	377 (15.0)	1.50 (1.27–1.76)		1.42 (1.05–1.91)	
**Previous pregnancies**						
Primigravidae	1289	254 (19.7)	1	<0.001	1	0.054
1–4 previous pregnancies	2489	368 (10.8)	0.49 (0.41–0.59)		0.65 (0.42–1.01)	
>4	1160	109 (9.4)	0.42 (0.33–0.54)		0.48 (0.25–0.90)	
**Mother's age**						
<20	1267	260 (20.5)	1	<0.001	1	0.016
20–34	3121	317 (10.6)	0.44 (0.37–0.52)		0.52 (0.34–0.82)	
>34	559	56 (10.0)	0.43 (0.32–0.59)		0.63 (0.28–1.44)	
**Period**						
> 2003–2006	3000	437(14.6)	1	<0.001	1	0.061
< 2009–2012	2223	225(10.1)	0.66 (0.56–0.78)		0.75 (0.55–1.01)	
**Study participant**						
No	2948	377 (12.8)	1	0.779	#	
Yes	2275	285 (12.5)	0.97 (0.83–1.15)			
**Mother's HIV status**						
Uninfected	1512	175 (11.6)	1	0.219	1	0.001
Infected	615	83 (13.5)	1.19 (0.90–1.58)		1.78 (1.27–2.50)	
**SES**						
poorest	430	49 (11.4)	1	0.71	1	0.88
2nd quintile	487	54 (11.1)	0.90 (0.61–1.31)		1.03 (0.64–1.65)	
3rd quintile	505	65 (12.9)	1.06 (0.74–1.53)		1.07 (0.67–1.73)	
4th quintile	488	53 (10.9)	0.98 (0.68–1.43)		1.14 (0.71–1.84)	
wealthiest	464	45 (11.2)	0.83 (0.56–1.23)		0.83 (0.50–1.38)	

* p value calculated through Wald Tests.

^ p value calculated through LR test.

# variable excluded in the model due to high collinearity with mother’s HIV status.

uOR = unadjusted odds ratio.

aOR = adjusted odds ratio.

### Mortality associated with preterm delivery and SGA during infancy

The overall neonatal mortality rate associated with preterm delivery (not SGA) was 599.3 (95% CI 224.9–1596.7) per 1000 CYAR and the infant mortality rate was 79.2 per 1000 CYAR (95% CI 35.6–176.3). Among preterm newborns regardless of the SGA status, the rates were 980.2 (95% CI 542.8–1769.9) and 136.1 (95% CI 84.6–218.9), respectively. The overall neonatal mortality rate among term SGA newborns was 289.0 (95% CI 137.8–606.1) and the infant mortality rate was 55.1 (95% CI 33.2–91.4). Among SGA newborns regardless of preterm delivery, the mortality rates were 427.5 (95% CI 242.8–752.7) and 76.6 per 1000 CYAR (95% CI 51.3–114.2) for the neonatal and first year of life period. The presence of both conditions (SGA and preterm birth) boosted both neonatal and infant mortality rates to 1299.8 (95% 541.0–3122.9) and 218.3 (95% CI: 113.6–419.5) per 1000 CYAR, respectively **(Table 4)**. No relevant differences on mortality rates were observed by sex of the infant.

**Table 4:** Mortality rates (deaths per 1000 CYAR) and hazard ratios (HR) of dying during the neonatal period and first year of life of different cohorts analysed.

	Subjects	Deaths	Time At Risk (CYAR)	Mortality Rate (Deaths per 1000 CYAR and 95% CI)	HR (95% CI)	p-value*
**MODEL 1**	** **	** **	** **	** **	** **	** **
**Neonatal Period**						
Term & non SGA	2336	21	193.9	108.3 (70.6, 166.1)	1	< 0.0001
Term SGA	296	7	24.2	289.0 (137.8, 606.1)	4.5 (1.6–12.6)	
Preterm & non SGA	84	4	6.7	599.3 (224.9, 1596.7)	13.0 (4.0–42.2)	
Preterm & SGA	53	5	3.9	1299.8 (541.0, 3122.9)	11.2 (3.0–42.1)	
**0–12 month Period**						
Term & non SGA	2341	64	2199.2	29.1 (22.8–37.2)	1	< 0.0001
Term SGA	296	15	272.2	55.1 (33.2–91.4)	1.9(0.9–3.8)	
Preterm & non SGA	84	6	75.8	79.2 (35.6–176.3)	3.7 (1.5–9.6)	
Preterm & SGA	53	9	41.2	218.3 (113.6–419.5)	8.9 (3.9–20.6)	
**MODEL 2**						
**Neonatal Period**						
Non Preterm	2635	28	218.4	128.2 (88.5–185.7)	1	< 0.0001
Preterm	147	11	11.2	980.2 (542.8–1769.9)	10.8(4.6–25.3)	
**0–12 month Period**						
Non Preterm	2640	79	2474.4	31.9 (25.6–39.8)	1	< 0.0001
Preterm	147	17	124.9	136.1 (84.6–218.9)	5.8 (3.1–10.6)	
**MODEL 3**						
**Neonatal Period**						
Non SGA	2420	25	200.6	124.6 (84.2–184.5)	1	0.0013
SGA	349	12	28.1	427.5 (242.8–752.7)	4.1 (1.7–9.6)	
**0–12 month Period**						
Non SGA	2425	70	2275.0	30.8 (24.3–38.9)	1	0.0016
SGA	349	24	313.5	76.6 (51.3–114.2)	2.5 (1.4–4.4)	

**HR:** adjusted Hazard Ratio. Cox multivariable regression model adjusted for: child sex, HIV status of the mother, number of previous pregnancies, period, mother age and socio economic status.

**CYAR:** children year at risk.

* P-value obtained through Wald tests.

Cox regression analysis adjusted for relevant variables is presented in **Table 4**. Both preterm birth and SGA conditions were independently associated with a higher hazard of dying during the neonatal period and infancy. During the first 28 days, preterm-non SGA delivery was associated with 13 times higher mortality rate (per unit time) compared to term deliveries not SGA (HR: 13.0, 95% CI 4.0–42.2), and term SGA was associated with about 5 times higher mortality rate (per unit time) when compared to the at term-non SGA group (hazard ratio 4.5 (95% CI: 1.6–12.6). The hazard of dying in the neonatal period for both preterm and SGA was higher when coexisting with each other. Mortality rates were still increased in the postneonatal period although of less magnitude, leading to lower hazard ratios associated to preterm and SGA compared to term-non-SGA babies. The hazard of dying the first year of life for both preterm and SGA was higher when coexisting with each other.

### Morbidity associated with preterm delivery and SGA during infancy

The incidence rate (IR) of outpatient clinic attendance was similar for the cohort of preterm-non SGA babies compared to that of babies born at term non-SGA, both in the neonatal and in the post-neonatal period (IRR 1.4, 95% CI: 0.9–2.4 and IRR: 1.0, 95% CI: 0.8–1.3 respectively). Likewise, there were no differences on outpatient attendances among term SGA newborns compared to those born at term non-SGA (**Table 5**). Most outpatient diagnoses in SGA and preterm infants were related to respiratory infections (29.2% and 20.1% respectively) followed by skin and conjunctivitis related visits (23.6% and 16.7%, respectively). However, no differences in the proportion of these diagnoses were observed in comparison to the at term non-SGA group.

**Table 5:** Incidence Rates (IR) of outpatient visits per 1000 CYAR and Incidence Rate Ratios (IRR) of visiting the outpatient clinic during the neonatal period and first year of life of different cohorts analysed.

	Subjects	Outpatient visits	Time At Risk (CYAR)	IR (per 1000 CYAR) and 95% CI)	aIRR (95% CI)	p-value*
**MODEL 1**	** **	** **	** **	** **	** **	** **
**Neonatal Period**						
non Preterm & non SGA	2336	447	193.9	2.3 (2.1–2.5)	1	0.5895
non Preterm & SGA	296	56	24.2	2.3 (1.8–3.0)	1.0 (0.7–1.4)	
Preterm & non SGA	84	20	6.7	3.0 (1.9–4.6)	1.4 (0.9–2.4)	
Preterm & SGA	53	10	3.9	2.6 (1.4–4.8)	1.1 (0.5–2.1)	
**0–12 month Period**						
non Preterm & non SGA	2341	7462	2199.2	3.4 (3.3–3.5)	1	0.6922
non Preterm & SGA	296	911	272.2	3.4 (3.1–3.6)	0.9 (0.8–1.1)	
Preterm & non SGA	84	251	75.8	3.3 (3.0–3.8)	1.0 (0.8–1.3)	
Preterm & SGA	53	155	41.2	3.8 (3.2–4.4)	1.1 (0.8–1.4)	
**MODEL 2**						
**Neonatal Period**						
non Preterm	2600	7876	2256.1	3.5 (3.4–3.6)	1	0.6884
Preterm	134	411	113.7	3.6 (3.3–4.0)	1.0 (0.9–1.2)	
**0–12 month Period**						
Non Preterm	2640	8379	2474.4	3.4 (3.3–3.5)	1	0.5676
Preterm	147	443	124.9	3.6 (3.2–3.9)	1.1 (0.9–1.3)	
**MODEL 3**						
**Neonatal Period**						
Non SGA	2420	467	200.6	2.3 (2.1–2.6)	1	0.9032
SGA	349	66	28.1	2.4 (1.9–3.0)	1.0 (0.7–1.3)	
**0–12 month Period**						
Non SGA	2425	7713	2275.0	3.4 (3.3–3.5)	1	0.5187
SGA	349	1066	313.5	3.4 (3.2–3.6)	1.0 (0.9–1.1)	

**aIRR:** adjusted Incidence Rate Ratio. Negative binomial regression model adjusted for: child sex, HIV status of the mother, number of previous pregnancies, mother age, period and socio economic status.

**CYAR:** children year at risk.

* P value obtained through Wald tests.

With regard to neonatal hospitalizations, these were more frequent only in babies with both conditions (IRR 3.5; 95% CI 1.5–8.1) compared to the term non-SGA group. The rate of hospitalizations was also increased during the entire first year of life in preterm (IRR: 1.7: 95% CI: 1.0–2.9) and preterm and SGA babies (IRR: 2.5; 95% CI: 1.4–4.5) (**Table 6**).

**Table 6:** Incidence Rates (IR) of hospitalizations (per 1000 CYAR) and incidence rate ratios (IRR) of being hospitalized during the neonatal period and first year of life of different cohorts analysed.

	Subjects	Hospitalizations	Time At Risk (CYAR)	IR (per 1000 CYAR) and 95% CI)	IRR (95% CI)	p-value*
**MODEL 1**	** **					
**Neonatal Period**						
non Preterm & non SGA	2336	113	193.9	0.6 (0.5–0.7)	1	0.0324
non Preterm & SGA	296	17	24.2	0.7 (0.4–1.1)	1.0 (0.6–2.0)	
Preterm & non SGA	84	7	6.7	1.1 (1.2–4.5)	1.6 (0.6–4.5)	
Preterm & SGA	53	9	3.9	2.3 (1.2–0.8)	3.5 (1.5–8.1)	
**0–12 month Period**						
non Preterm & non SGA	2341	507	2199.2	0.2 (0.2–0.3)	1	0.0082
non Preterm & SGA	296	74	272.2	0.3 (0.2–0.3)	1.2 (0.8–1.6)	
Preterm & non SGA	84	20	75.8	0.3 (0.2–0.4)	1.7 (1.0–2.9)	
Preterm & SGA	53	22	41.2	0.5 (0.4–0.8)	2.5 (1.4–4.5)	
**MODEL 2**						
**Neonatal Period**						
non Preterm	2635	132	218.4	0.6 (0.5–0.7)	1	0.0176
Preterm	147	17	11.2	1.5 (0.9–2.4)	2.2 (1.2–4.4)	
**0–12 month Period**						
Non Preterm	2640	583	2474.4	0.2 (0.2–0.3)	1	0.0027
Preterm	147	45	124.9	0.4 (0.3–0.5)	1.9 (1-3-2.9)	
**MODEL 3**						
**Neonatal Period**						
Non SGA	2420	120	200.6	0.6 (0.5–0.7)	1	0.2767
SGA	349	26	28.1	0.9 (0.6–1.4)	1.4 (0.8–2.3)	
**0–12 month Period**						
Non SGA	2425	527	2275.0	0.2 (0.2–0.3)	1	0.088
SGA	349	96	313.5	0.3 (0.3–0.4)	1.3 (1.0–1.8)	

**aIIR:** adjusted Incidence Rate Ratio. Negative binomial regression model adjusted for: child sex, HIV status of the mother, number of previous pregnancies, mother age, period and socio economic status.

**CYAR:** children year at risk.

* P value obtained through Wald tests.

## Discussion

This is one of the few studies carried out in sub-Saharan Africa evaluating the impact of both prematurity and small for gestational age births on mortality and morbidity during the first year of life. Information available on the health impact of these two conditions mostly focus on the neonatal period and derives from high or middle income countries. These findings show that neonatal and infant mortality rates are remarkably higher during the neonatal and post-neonatal periods in both preterm and SGA babies compared to babies born at term and non SGA. However, preterm birth is associated with even higher neonatal and infant mortality, almost two fold, compared to SGA without prematurity. This information is fundamental to guide preventive and management measures.

The analysis has been done using different statistical models in order to allow for different but important interpretation of the results, namely, the evaluation of preterm and SGA births as independent conditions (model 1, main model), but also the evaluation of these conditions without considering the presence of the other (model 2 and 3). Prematurity was associated with almost a 13 and 4 fold-increased risk of dying during the neonatal and the postneonatal period, respectively. Small for gestational age on the other hand, was associated with a lower risk of death compared to preterm births in all models, in line with findings from other studies[10,24]. Since SGA definition is based on a statistical approach, babies with SGA might or might not be associated with a specific morbid condition during pregnancy, and they could be considered healthy children having no adverse consequences or complications during infancy[25].

Our results on mortality rates associated with preterm and SGA in the neonatal period are in line with those published in a recent pooled country analysis for low and middle income countries[26]. It has been reported that the mortality rate among preterm births is almost two fold increased during the second year of life [11]. These results confirm that the increased risk is mostly concentrated during the neonatal period as it has been described long time ago by Barros and colleagues [10]. In the analysis (model 1) for the post-neonatal period (data not shown) an increased mortality rate associated with preterm delivery or SGA is not observed. However, when analysing these conditions without taking into account the presence of the other (models 2 and 3), an increased mortality during the postneonatal period is observed (around three fold for preterm and 1.8 fold for SGA babies). This apparent discrepancy could be explained by the presence of confounding, that is, in the preterm group there are many SGA babies, distorting the independent association of prematurity with mortality. Likewise, the same confounded association would occur when estimating mortality among SGA babies.

Interestingly, the results on morbidity seem to be contradictory with the mortality findings. It seems that neither prematurity nor SGA births are associated independently with higher rates of hospitalization during the neonatal period compared to those term non-SGA. Model 1, only shows an increased risk of hospitalization when both are present (IRR 3.5, 95% CI: 1.5–8.1), but not when they are analysed separately. Moreover, we did not observe an increased risk of outpatient attendances in the preterm or SGA cohort for any of the periods. This could be due to several reasons. First, small numbers of hospitalizations and outpatient visits in the preterm and SGA cohorts might have hindered the chances of finding this association if it does indeed exist. Second, morbidity due to mild conditions might be similar between the groups, and increased morbidity risk might only be associated with severe conditions, which might be best reflected when analysing hospitalization risk of both SGA and preterm delivery. Third, in this area of southern Mozambique many children are first taken to the traditional healer when they are sick. If the potential health problems associated with preterm and/or SGA are severe, children might not be taken to the formal health system before they die. Thus, morbidity surveillance based on outpatient or inpatient attendances might be an underestimate of the true morbidity burden associated with these conditions.

These findings underscore the need to identify these conditions early enough in order to implement interventions aimed at increasing the level of care, and ultimately survival. However, with currently available strategies, there is a broad room for improvement in the field of prevention, which should focus in targeting the known risk factors, including: preconception counselling and family planning; health education programs aimed at prevention, early diagnosis and treatment of infections before and during pregnancy; increased control of conditions such as diabetes, hypertension, anaemia, before and during pregnancy; close monitoring of nutritional status and mental health of the mother, as well as implementation of best practices in assisted reproduction (which includes training to all health care workers involved)[27]. Although in our setting the rate of induced labour before week 37 is negligible, other settings should closely monitor and potentially reduced these practices, as well as rates of caesarean section.

This study has several limitations. First, gestational age was measured through indirect methods based on postnatal examination of the newborn (Dubowitz test, Ballard score). Although both methods have been validated and are broadly used in low income countries[28], the accuracy, agreement and reproducibility of these methods have been questioned[29]. The Dubowitz test might underestimate GA in SGA and term infants[30], although it could also overestimate GA in very preterm infants (<33 weeks)[31]. Some assessments have also questioned the accuracy of the Ballard score[29]. If any of the methods would underestimate the GA, the prevalence of preterm birth could be slightly overestimated and mortality and morbidity rates underestimated in comparison to the at-term non SGA group. In addition, in order to calculate gestational age, children had to survive the first hours of life and be hospital delivered, thus some deaths occurring before gestational age was assessed were not included. This would certainly underestimate the prevalence of prematurity and small for gestational age (but also the mortality risk associated with these conditions). Lastly, the fact that mothers have participated in a research study might have underestimated the true prevalence of preterm birth in this setting. It could be thought that this could have also positively contributed to better health outcomes in the first year of follow up. However, a majority of children with available prospective data belonged to the mentioned studies, thus, we believe our measures of effect are not biased in those born from study participants. If so, our findings would represent a conservative estimate of the true mortality and morbidity rates.

## Conclusions

In conclusion, these results contribute to the evidence on the increased risk of mortality and morbidity associated with preterm and small for gestational age births in rural Africa. This increased risk is much higher for preterm births than for SGA without prematurity and appears to be concentrated within the neonatal period. Routine assessment of birth weight and gestational age at birth, and identification of these conditions should prompt interventions aimed at increasing the level of care among these high-risk newborns and improve survival.
